# Sociocultural factors that influence the prevention of malaria in Ohangwena region, Namibia

**DOI:** 10.4102/phcfm.v14i1.3524

**Published:** 2022-08-30

**Authors:** Selma I. Uushona, Jacob A. Sheehama, Hermine Iita

**Affiliations:** 1 Department of Community and Mental Health, Faculty of Health Sciences, University of Namibia, Oshakati, Namibia; 2 Department of Microbiology and Chemistry, Faculty of Health Sciences, University of Namibia, Windhoek, Namibia; 3 Department of Public Health, Faculty of Health Sciences, University of Namibia, Oshakati, Namibia

**Keywords:** sociocultural factors, prevention, malaria, Ohangwena region, Namibia

## Abstract

**Background:**

Namibia is undergoing an epidemiological transition after decline in local transmission of malaria, and the country is now in a position to move towards eliminating local transmission by 2030. However, malaria prevalence cannot be adequately explained from medical and modern prevention points of view alone. The persistence of malaria might appear as a result of not recognising sociocultural factors that seem useful in the prevention of malaria, Hence, studies on sociocultural factors are limited.

**Aim:**

The aim of this study was to describe the sociocultural factors that influence the prevention of malaria in Ohangwena region.

**Setting:**

The study was conducted in Ohangwena region of northern Namibia.

**Methods:**

This study was a cross-sectional study and a mixed methods, convergent parallel design was employed.

**Results:**

The major theme revealed that traditional prevention methods of malaria are widely available in rural communities. The best accepted traditional prevention methods include tumbleweed, bitter bush and animal dung. Quantitative findings indicated that 67.0% of participants felt that nets are expensive. Key barriers included the long distance to access health facilities (29.1%), long waiting times (25.8%) and the lack of money to pay for services and transport (22.5%).

**Conclusion:**

The limited access to and cost of Western prevention methods minimise protection because of priority and resource allocations, but it could be mitigated with the use of locally available traditional prevention practices used for many years in curbing malaria. There is a need to create awareness about socioculturally congruent malaria care.

**Contribution:**

This study has revealed the need to combine standard prevention with traditional prevention practices in the fight against malaria, and it intensified research focusing on interventions that address sociocultural factors for the prevention of malaria in endemic regions. In addition, part of the novelty of the study is establishing the need to test the efficacy of traditional practices used.

## Introduction

Malaria remains a major public health concern worldwide.^1,2,3^ Sub-Saharan Africa is particularly affected by malaria, accounting for nearly 90% of malaria cases in Africa. Namibia is a sub-Saharan country and one of the four Southern African Development Community (SADC) countries that have united in a campaign to eliminate malaria from the region.^4^ The SADC strategy is aimed at eliminating malaria in Botswana, Namibia, South Africa and Eswatini.^5^ Namibia is recognised as one of the four countries in Southern Africa that is positioned to reorient its malaria programme from control to an elimination programme.

Namibia focuses on standard prevention methods. These modern interventions include quality indoor residual sprays, parasitological diagnosis such as microscopy and increasing access to rapid diagnostic tests, the introduction of new and effective artemisinin-based combination treatment and personal protection, including the use of long-lasting insecticide-treated nets and mosquito coils.

Despite the availability of Western interventions, Namibia is not exempted from the burden of malaria, which differs from region to region. About 70% of the population lives in areas where there is some risk of malaria transmission. This burden was experienced in the 10 regions where malaria is endemic. Between 2014 and 2021, the country reported a malaria incidence of greater than 1 per 1000 population in 12 district regions out of a total of 14 regions, in contrast to the expected reduction of less than 1 per 1000 population. Some regions such as Ohangwena, Kavango East, Kavango West, Zambezi Oshikoto, Oshana, Omusati and Kunene recorded malaria incidences above 5 per 1000 population.^6^

The trends of malaria transmission are alarming, yet the prevention interventions that have been utilised focus more on medical and modern Western practices and points of views. The country adopted a national strategy to eliminate local malaria transmission, an intervention known as surveillance guidelines for malaria elimination.^6^ The use of medical treatment, diagnosis and Western prevention practices has been seen as the best weapon that brought about reduction in malaria transmission. However, the disease is complex, affects all people and its prevention is influenced by many factors. Among these are the social and cultural factors that influence malaria transmission and prevention. Therefore, malaria incidence cannot be adequately explained from medical and modern Western prevention points of view alone. The persistence of malaria might appear as the result of not recognising the sociocultural factors that seem useful in the prevention of malaria. Studies on these sociocultural factors are limited. In addition, continued persistence of the disease appears to be largely because of economic factors, human behaviours, traditional beliefs and livelihoods that are at variance with the Western prevention and control strategies, such as the use of long-lasting insecticide-treated nets and indoor residual sprays.

Understanding sociocultural factors is essential to support the elimination strategy of local transmission in the region. Culture underlies health care delivery at client, provider and system levels because it is the foundation for expectations, interactions and meanings of care.^7^ As communities grapple with the realities of human morbidity and mortality, issues of culture come into focus in ways that heighten the relevance of sociocultural practices at each level of these experiences.

Several researchers have documented studies on sociocultural factors from other countries, such as Manu et al.^8^ in Ghana, Jombo et al.^9^ in Nigeria, Das et al.^3^ in India and Dunn^10^ in Tanzania. These authors address the contribution of the Western malaria prevention and control strategies, respectively. However, Namibia has limited available documented data for sociocultural factors and practices, even though the country has good documented information of the Western malaria prevention strategies incorporated in the integrated primary care services. These integrated primary care services are oriented towards medical prevention interventions, with limited focus on sociocultural factors. As a result, these primary care services have no documented traditional practices for the prevention of malaria. It is difficult to accept the recognition of sociocultural practices in the prevention of malaria without endorsement in the *National Health Act of 2015*.^11^ This study goes beyond these parameters by describing and bringing to the forefront the sociocultural factors that influence the prevention and control of malaria among the rural communities in the Ohangwena region in Namibia.

This research aims to identify and describe the sociocultural factors that influence the prevention of malaria in rural communities in the Ohangwena region. The study was conducted in four phases. However, this article focuses only on phase 1. Phase 1 involved a situational analysis with three objectives: (1) to identify and describe the sociocultural factors related to malaria prevention and control practices in the selected area; (2) to assess the knowledge, attitudes and practices (KAP) of participants; and (3) to explore and describe the perceptions of rural communities regarding sociocultural factors and how these influence traditional practices and standard malaria prevention in the Ohangwena region. In this phase the following variables were utilised: (1) participants’ background, (2) knowledge, (3) malaria prevention practices, (4) attitudes towards traditional and modern interventions, (5) net acquisition and ownership, maintenance care and treatment, (6) access to care, (7) health-seeking behaviours, (8) barriers to using health facilities, (9) environmental and livelihood factors that facilitate the prevalence of mosquito bites, (10) knowledge of other methods used to prevent malaria and (11) perceptions of traditional prevention and standard malaria prevention practices. The central point of this study was to provide guidance on how to bridge the knowledge gap in social and cultural factors in the training of health care workers that would result in sustainable progress that supports the national strategy towards the elimination of the local transmission of malaria.^4,6^

## Research methods and design

Evidence-based research generates ways of bridging the gaps identified in the literature, and thus it broadens and refines existing knowledge through various innovations. The current study used an evidence-based approach.

### Study design

This study used a convergent parallel mixed methods design in which quantitative and qualitative methods carried equal weight.^12,13,14^ This design was selected to assist the researcher to obtain data about people’s lived experiences and describe the daily practices of malaria prevention among rural communities. Quantitative, descriptive, observational and qualitative exploratory phenomenological and contextual approaches^15^ as well as theory development approaches^16^ were used, as outlined below.

The quantitative data comprised numerical values for each respondent and described the data in the form of grouped data, graphical representations and frequency tables that assisted in quick observation of data distributions.^14^ A descriptive design was needed for the provision of accurate data, fact-finding among variables and the interpretation of findings to promote comparison and the identification of relationships.

An observational design was chosen to capture events as they occurred in their natural setting. Internal and external factors were observed without disturbance from the researcher, and a checklist was used during direct observation.

A qualitative design was used to explore and validate the phenomenon of how sociocultural factors influence malaria prevention practices in the selected rural communities. The narrative and visual information was obtained to make sense out of participants’ experiences and perceptions of sociocultural practices regarding the prevention of malaria. In-depth face-to-face interviews with heads of households and focus group discussions (FGDs) with health extension workers (HEWs) were conducted.

An exploratory design assisted the researcher to obtain insight and understanding of rural communities and their perceptions towards sociocultural factors from participants’ points of view. Phenomenology is concerned with discovering reality by probing to determine the root of the problem in a meaningful manner. The triangulation of various methods and tools allowed for in-depth insight into the research problem.

A contextual approach was needed to describe and interpret the interaction of shared values, sociocultural practices and traditional behaviour in shaping the lived experiences of participants and the meaning attached to their daily experiences of the sociocultural prevention of malaria. The context where the study took place is the Ohangwena region, and interviews were conducted in the home environment of the participants. This helped to minimise external influences and disturbance and promote trust and the collection of valid information.

The study was cross-sectional, as it was conducted in a single round of data collection to investigate what currently existed.^17^ The data were collected at the same time and the results were merged with the purpose of comparing to produce a valid conclusion.

**FIGURE 1:**
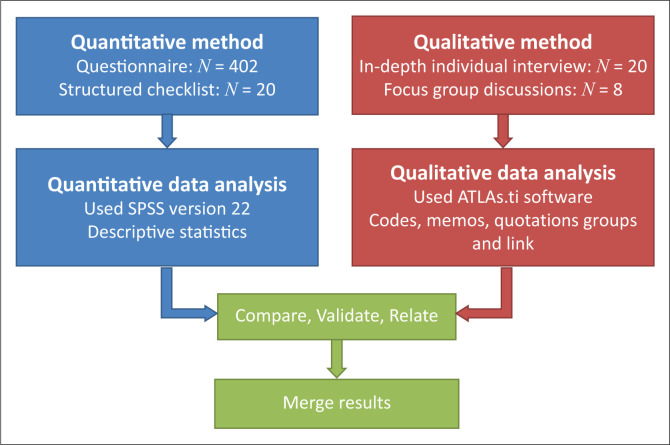
Implementation of the convergent parallel mixed methods design used in this study.

The above research approach assisted the researcher to obtain rich information of rural peoples’ lived experiences, apply principles of social justice and obtain a complex holistic picture^14^ in order to obtain a clear understanding of socioculturally congruent malaria care in the selected rural communities. The research methodology is summarised in Figure 1.

### Setting

The study was conducted in the Ohangwena region of northern Namibia. The environment was natural and familiar to participants as the completion of the questionnaire occurred in public gathering places and homes, while direct observation using a checklist and face-to-face interviews took place in the participants’ homes. The FGDs were held in clinics. The study was limited to rural communities and rural clinics in Ohangwena region of northern Namibia.

### Population and sampling

The target population was 220 684, and the sample size was determined by using the Yamane formula.^18^ The calculation determined the minimum required sample size to be 399, but the researcher managed to get data from 402 respondents. The study used multiple sampling methods in order to collect rich data of high quality.^14,17^

The quantitative component of the study used a probability sampling method, which was guided by the fishbowl technique. Participants were randomly selected using a simple random sampling with replacement to facilitate equal selection of participants and promote generalisation of data to the study population.

The qualitative component employed an exploratory and descriptive approach to obtain clear insight into the perceptions of heads of households and HEWs on sociocultural and traditional practices in relation to malaria prevention. Nonprobability sampling method was used, in particular purposive and snowball sampling. Purposive sampling was used to select HEWs based on the inclusion criteria that participants were knowledgeable and capable of providing true information. In selecting the heads of households, snowballing was used to select participants to be interviewed because of easy referral of participants who met the desired characteristics and were willing to participate in the study. The participants were selected based on their experiences of traditional practices and indigenous knowledge and were expected to offer comprehensive responses that generate good insight and better understanding to enrich findings. Participants were selected based on deployment at rural clinics where one or more positive rapid diagnostic test cases were identified. Furthermore, clinics where no HEWs were employed and no malaria cases were detected during the period of the study were excluded from the study. Health centres and district hospitals were excluded because these facilities act as referrals from clinics and are located in towns or settlements with better resources. The participants took part voluntarily in the study.

The total number of HEWs in Ohangwena region was 188 at the time of the study, and heads of households were selected from 37 404 rural houses. However, the provided number was not used to determine the sample size for qualitative data as there is no rule that specifies the number for sample size in purposive and snowballing, especially on nonprobability sampling. In addition, the determination of sample size in qualitative research is still a dilemma, and results are supported by rigorous processes and by squeezing as much rich and thick, descriptive information from participants as possible in order to obtain sufficient information.

The sample size for the qualitative components was determined by data saturation. Data saturation was reached at a point where no new information emerged and the results observed came to a point of repeated information, which came to a total number of eight FGDs and 20 individual interviews.

A total of 20 in-depth face-to-face interviews were held with heads of households, while eight FGDs were conducted with HEWs, each involving 6–10 participants. There is no rule to determine a specific number, as sample size is not easy to predetermine in qualitative research.^14,17^

### Data collection tools

The data were collected from October 2017 to January 2018 through survey questionnaires and interviews with different sources to allow for comparisons and cross-checking.

In the quantitative component, data were collected using a questionnaire (*n* = 402) and a checklist (*n* = 20) that were used for direct observation. The questionnaire was developed with closed- and open-ended questions. The questionnaires were self-delivered by the researcher and checked for completeness immediately upon collection from the participants to reduce poor return. The checklist contained a predetermined response of ‘yes’ and ‘no’.

In addition, semistructured interviews using an interview guide were used to generate narratives on the influence of sociocultural factors on malaria care (*n* = 20) in order to obtain rich, valid and thick descriptions from each participant that helped the researcher to understand the perceptions and learn from the opinions, ideas and beliefs of rural communities regarding the sociocultural prevention of malaria. In-depth face-to-face interviews were conducted with heads of households. Eight FGDs were also held with HEWS. In addition, data that were not captured easily with questionnaires and interviews were captured through direct observation. The following factors were observed: the participant’s home environment, the type of house and construction materials used, the means of communication and the type of malaria prevention methods used. Both face-to-face interviews and FGDs were recorded with an automatic battery-operated recorder to minimise recording failure. All recorded sessions were transcribed verbatim, and data were coded to generate themes and subthemes. The researcher also took field notes to supplement the recorded information.

### Measures for ensuring the quality of data

All instruments developed by the researcher were evaluated by her supervisors and external experts. The content of the instruments was aligned with the objectives of the study. The instruments were pilot tested to determine feasibility and were further refined during data collection as changes were incorporated. This was done to maintain consistency or reliability in measurements.

Validity was enhanced by the use of the mixed methods approach or methodological triangulation; content validity was promoted by reference to the study objectives as data gathered from interviews were grouped into themes and subthemes and face validity was checked and confirmed. All tools were piloted to increase stability. Collaboration with the researcher’s supervisors, seminar presentations and peer examination also served to validate the content.

Trustworthiness was confirmed by using Lincoln and Cuba’s four criteria, namely credibility, transferability, dependability and confirmability.^19^ Credibility was enhanced as the researcher met the participants face-to-face; there was prolonged engagement and triangulation of data. Data were transcribed verbatim, thus also enhancing authenticity. Transferability is applicable as the researcher produced a thick description of information that provided an understanding of the lived experiences of rural communities and how they deal with malaria using traditional practices in their daily lives. The data that were obtained from the direct quotations of the participants, conducting direct observation and tape recording, were used to support the findings of this study. This assists other researchers in terms of replication.

Confirmability was established as data were derived from the participants. The recorded data, transcripts, filed notes, themes and subthemes were reviewed by supervisors and experts from the University of Namibia. Confirmability was improved by maintaining high standards through external audit.

### Data analysis

The researcher coded each item before entering it into the Statistical Package for the Social Sciences (SPSS) version 22 for further analysis. The use of quantitative data helps in the generalisation of results to the rest of the rural population in the Ohangwena region. Descriptive and inferential statistics were used. After data cleaning, the data were described according to five variables: demographic information; knowledge; attitudes towards and practices of malaria prevention in relation to both sociocultural, traditional practices and standard prevention; livelihood; and social and environmental factors, as well as barriers to accessing health care.

The study findings were presented and interpreted using frequency and contingency tables, cross-tabulation, graphs and charts. The qualitative data from the FGDs and in-depth interviews were transcribed verbatim and analysed using ATLAS.ti to generate themes, subthemes and categories.

### Ethical consideration

Approval to conduct the study was obtained from the University of Namibia’s Research and Publication Committee. Permission to enter the region was obtained from the Ministry of Health and Social Services (reference number 17/3/3 SU). Additional permission was obtained from the Health Director of Ohangwena to start with the process of data collection. Important ethical principles that guided this study included all participants giving oral consent. No personal details were required in order to respect the dignity and maintain the confidentiality of participants. All the completed tools were kept in a locked suitcase stored in a locked room, and access to data was strictly controlled for the duration of the study period. Participants were treated equally and had freedom to withhold their views if they felt uncomfortable. No inducements were used, and participation was voluntary. Care was taken to avoid any physical, social, emotional and psychological harm.

## Results

The descriptive results were presented in tables, graphs and charts. The narrative results were presented as verbatim quotations as well as the formulation of themes and subthemes. As the research was a mixed methods study, quantitative and qualitative data were merged using a side-by-side comparison approach, which is done at the end of the results section.

### Quantitative results

A total of 402 respondents completed the questionnaire. This study revealed that 72.3% of the respondents were women, compared to 27.7% male respondents. The male respondents, culturally, do not generally participate in household health activities, as these roles are associated with women. Bed nets initially targeted women and not men; therefore, when information was needed about malaria, men linked malaria to women and children. The majority of men refused to participate and referred the researcher to women available in the households. The way mosquito nets were distributed in Namibia in the past was by targeting women, children and elders. The targeting programme created an impression among men, irrespective of age, that net use was linked to women. Therefore, their use is associated with vulnerability. The study findings revealed that ownership of mosquito nets is more prevalent among women of child-bearing age (68.3%) and elderly people (61%).

**FIGURE 2:**
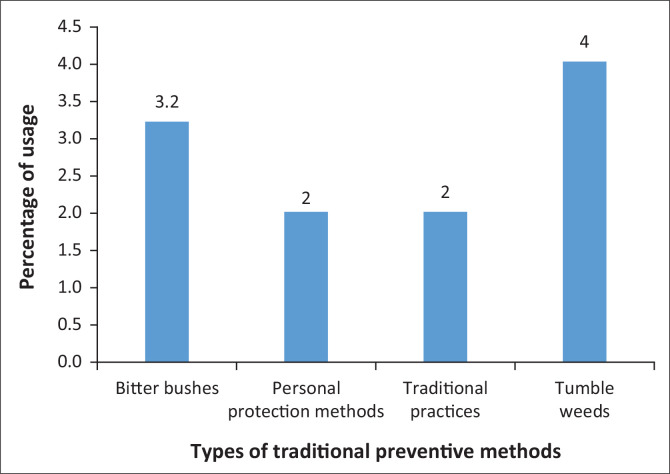
Traditional methods to prevent malaria.

In addition, the mosquito nets are often acquired through donation (66.4% from various organisations, both state and nongovernmental organisations), compared to 13.6% of respondents who can afford to buy nets. It is clear that the majority of rural community members are not able to afford mosquito nets (41.7%). The study findings revealed, among other factors, that rural communities perceived that mosquito nets are expensive (68.7%) and some people are not comfortable with using nets (12.7%), whereas 9.3% indicated that nets are not a priority for them and they have fear of itching. This result of mosquito nets not being a priority was supported by net owners who did not sleep under mosquito nets. Malaria prevention in the Ohangwena region has been focused on modern accepted prevention methods; hence, it is necessary to utilise indigenous knowledge when introducing methods for care and prevention of malaria in resource-limited settings. Despite the availability of mosquito nets, these were found kept in the sleep rooms unused, wrapped in the cover. This was revealed by 46.4% of participants who slept under a bed net a night before the study, in comparison to 53.6% who did not use one. Instead, some members of the rural community use other methods, apply these in the form of burning or fumigation, rubbing a mixture on the skin or hanging and inserting leaves in the roof inside the sleeping rooms to repel mosquitoes. The study results indicated that there are a few rural people who have access nets but instead choose to use traditional practices and indigenous plant resources (29.4%). This included the use of other traditional prevention practices such as burning marula fruit skin, burning elephant dung and egg boxes and covering the body with blankets, wearing long-sleeved clothes and covering the body with a cloth (18.2%). Other traditional methods include fire (10.6%) and sociocultural practices (29.4%), for example, using animal dung or the leaves of selected plants such as *eingamwe*, Madagascar periwinkle, tumbleweed (4%), *tamboti* wood, bitter bushes (3.2%) and blue bush (2%). These traditional prevention practices are presented in Figure 2.

These preferences and beliefs might be because of technical issues around the use of mosquito nets and lack of income to afford nets. Knowledge of cleaning and maintaining the mosquito nets is also a challenge. There is limited knowledge regarding the type of soap used for washing mosquito nets, as 37.8% of participants only had knowledge that the Sunlight^®^ bar soap is recommended for net cleaning. The majority (53.4%) indicated using powder soap and hanging the net in direct sunlight (51.2%) for drying, which may reduce the efficacy of the insecticide and increase the likelihood of exposing members of the household to mosquito bites.

**FIGURE 3:**
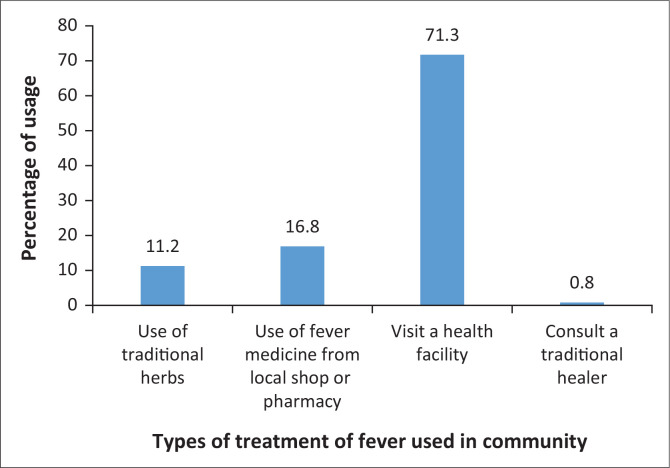
Treatment of fever in the community.

Respondents stated that they use various means to treat fever. Figure 3 shows that 71.3% of respondents visited a health facility, 16.8% found medication from a local shop or pharmacy, while 11.2% used traditional herbs. Only 0.8% of respondents reported consulting a traditional healer for the treatment of fever.

When participants’ knowledge of the severity of fever was probed, the following symptoms were indicated: vomiting (71%), yellow eyes and passing loose stools (45.8%) and loss of alertness, change in behaviour and confusion (27.1%). When it came to decision-making, any adult person (64.3%) available at home could make decisions for a feverish sick member to seek treatment.

**FIGURE 4:**
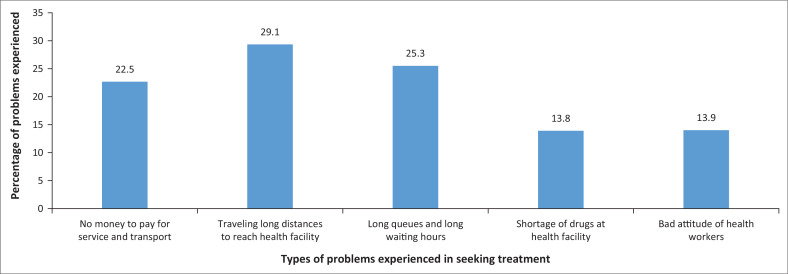
Problems experienced in seeking treatment.

It was important to ask about the barriers to seeking malaria treatment. Figure 4 shows key barriers including the long distance to access health facilities (29.1%), long waiting times (25.8%) and the lack of money to pay for services and transport (22.5%). These hindrances involved economic, infrastructural and time management factors. These factors may serve as barriers to timely treatment.

Some of the respondents (22%) expressed that they lack knowledge of on-time treatment, by indicating 4–12 days, in contrast to three days, which is the maximum time allowed for early reporting and diagnosis in the guidelines of the Ministry of Health and Social Services in Namibia.

This lack of knowledge is aggravated by social environments that promote the thriving of mosquitoes. This relationship of malaria transmission and social environments is supported by the way rural communities interact with the environment for their daily survival and livelihood. Approximately three-quarters of participants (73.5%) have livestock living in close household surroundings. The study findings indicated that 78.8% of livestock kraals are found at a distance of 50 m – 100 m from the households. Moreover, 10.2% of participants had their animal kraals attached to their households and 11% had kraals located more than 100 m away from households. Livelihood and human survival also influence mosquito bites and malaria transmission by the location of households in relation to millet fields, forest topography and points of water sources.

**FIGURE 5:**
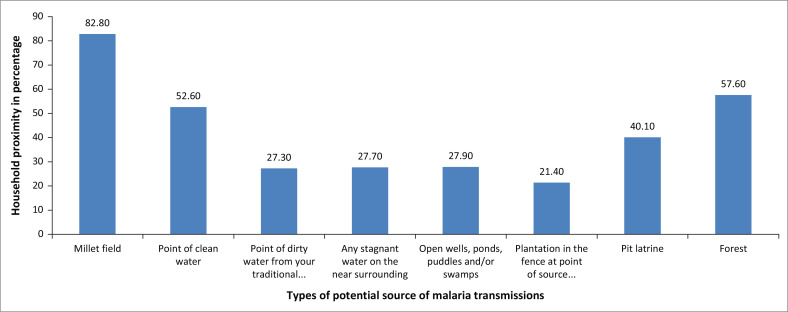
Proximity of household to key points of potential malaria transmission.

Figure 5 shows that the majority of the households (82.8%) reported that their houses were built close to a millet field; 57.6% reported that their houses were built near a forest; 52.6% reported that their houses were built close to a point of clean water, while 40.1% reported that their houses were built near a pit latrine. More than a quarter (27.3%) of respondents reported that their houses were built near a point of dirty water, and 27.7% had their houses built near stagnant water points. In addition, 27.9% reported to have their houses built near open wells or swamps and 21.4% reported that their houses were built near plantations. The environment is in favour of mosquito breeding and hiding sites. Despite millet, the source of water in rural areas is from wells and open flat surfaces that collect water during the rainy season.

### Qualitative results

These results were generated from 20 in-depth interviews with heads of households and eight FGDs with HEWs. Verbatim transcription of interviews was done to generate themes, subthemes and categories. The study generated six themes, but for the purpose of this article, only four themes and 20 linked subthemes are presented in Table 1.

**TABLE 1:** Themes and subthemes generated from qualitative data.

Themes	Subthemes
Theme 1: Participants perceived malaria as a disease of ‘others’	1.1 Lack of interest to participate in malaria activities among men.
	1.2 Men perceiving malaria as a disease for others, such as women, children and elderly people.
	1.3 Men spent longer periods at social gatherings and shebeens without using protective measures. (Shebeens are informal businesses where people buy alcoholic drinks and other commodities in rural areas or informal settlements.)
Theme 2: Participants perceived that their economic status influences their capabilities to manage malaria at the community level	2.1 Lack of money influences decisions on net use.
	2.2 Many adults do not have enough money to purchase mosquito nets for the whole family.
	2.3 Adults only purchase mosquito nets for owner use.
	2.4 Lack of income-generating activities.
	2.5 Production of marula oil is not undertaken as this requires considerable energy.
Theme 3: Participants perceived that mosquito nets are generally not used	3.1 Bed nets are difficult to use in traditional sleeping huts.
	3.2 Bed nets are kept unopened; participants wait for special events, birth of newborn baby.
	3.3 Bed nets are kept unopened; participants wait for the adult who works far from home.
	3.4 The net is connected to assets.
	3.5 Mosquito nets were not perceived as a priority.
	3.6 Net use is linked to the position status of the member in the family hierarchy.
Theme 4: Participants perceived that there are barriers to access malaria care	4.1 Travel long distances to access health facilities.
	4.2 Lack of money.
	4.3 Poor infrastructure.
	4.4 Limited access to social media information.
	4.5 Waiting long periods at overcrowded health facilities.
	4.6 Health extension workers not able to reach many households because of long distances and large numbers of allocated houses per HEW.

HEW, health extension worker.

A summary of the narratives is discussed as follows:

It was observed that there was a lack of interest in participating in malaria activities among men, which was inferred by the lack of initial involvement in malaria prevention activities. Firstly, limited participation in and ownership of malaria preventative activities among men contributed to men perceiving malaria as a disease for others, such as women, children and elderly people. This resulted in men spending longer periods at outdoor social gatherings and shebeens without using protective measures.

Secondly, the lack of money influences decisions on net use. Many adults do not have enough money to purchase mosquito nets for the whole family, so they only purchase mosquito nets for their own use. Some income-generating activities such as the production of marula nut oil are not undertaken as they are time-consuming and energy draining. In addition, the lack of employment is another problem experienced by the selected rural communities.

Furthermore, bed nets are often not used and are kept unopened, as people wait for special events such as the birth of a baby. Some people are not always at home, but their nets are kept for them and no one uses them simply because they are regarded as assets. As a result, young children and adolescents sleep without any protection. This also depends on the position of a child in the household, as children have little claim to expensive protective goods such as bed nets, and children from 5 to 17 years of age have low status in the family hierarchy.

Finally, respondents stated that they travel long distances to access health facilities and have no money to pay for transport and better services. Other challenges revealed were poor infrastructure with reference to roads, low network coverage, limited social media and information and technology. The lack of access and long distances to health services deter people, which encourage some people to promote traditional practices for malaria prevention and home-based healing practices in secrecy.

Another barrier was the problem of waiting long periods because of overcrowded health facilities and poor time management on the part of staff members at these facilities. Bed nets are difficult to use in traditional sleeping huts, resulting in nets not physically hanging at the site of the bed. Furthermore, mosquito nets were not perceived as a priority and the rural communities experienced pressing needs. The available resources were used for human food, an indication that participants could not afford both mosquito nets and food for people and domestic animals.

Long travelling distances also affected the HEWs; they were not able to cover all households in a short time because of the distances and the incompatible ratio of a large number of allocated households per HEW, which compromised service delivery to many rural communities.

## Discussion

Both the quantitative and qualitative findings will be discussed together. Table 2 presents a summary of the key findings side by side for comparison purposes. The indicator strategy shows whether the findings are similar or divergent.

**TABLE 2:** Summary of key findings from the quantitative and qualitative data.

Influence of some demographic information on knowledge pertaining to modern prevention and traditional practices of malaria prevention	
Quantitative findings	Qualitative findings
Bed net acquisition was associated with donation and free distribution from the Ministry of Health and Social Services.	Nets are received from mass campaigns and health facilities.
Majority of bed net usage is found among women who are breastfeeding, caregivers with infants and elderly women.	Patterns of bed net usage were observed among caregivers for infants and children under the age of five years, mothers and grandmothers.
Nets are distributed by NGOs free of charge.	Nets distributed free of charge from Trans-Kunene Initiative and HEWs.
Initial nets were given to women, children and elders.	Net distribution targeted women, children and elders.
**Bed net ownership and usage**	
Age and gender play a role in bed ownership and usage.	The role age plays has influence on bed ownership and usage.
Bed net usage is found among women who are breastfeeding, caregivers with infants and elderly women.	Bed net usage was observed among caregivers for infants and children under the age of five years, mothers and grandmothers.
**Influence of social status, cultural acceptance, economy and educational status on malaria prevention**	
Lack of employment.	Lack of working opportunities and development in the region.
Bed nets are expensive.	Mosquito nets are expensive.
Mosquito net was not a priority.	Available money was used for buying food and for other pressing needs.
	Able to afford mosquito net for own use but not for the whole family.
The level of education influences the understanding of mosquito net usage and purchasing.	Literacy increases awareness and influences the usage of bed nets.
**Low level of knowledge among HEWs on traditional practices**	
Participants are advised to always use bed nets.	Sleeping under treated bed nets was seen as the best method in malaria prevention.
	Limited autonomy
	HEWs demonstrated low confidence, low self-esteem and negative attitudes towards the use of traditional practices.
	HEWs sometimes discouraged people from using traditional methods.
Traditional methods for malaria prevention are widely available but limited in use.	Participants felt powerless over life choices.
	The imposing ideas of HEWs undermine the rural community members’ ability to make their own decisions in choosing suitable methods available to prevent malaria.
**Environmental factors and livelihood**	
Three quarters of participants have livestock living in close household surroundings.	–
Houses were built close to a millet field, forest and a point of clean water.	
**Treatment of fever**	
Treated at health facility and self-treatment.	Refer by HEWs to clinic.
Use of traditional herbs and consulting a traditional healer.	Treatment received from clinic.
	Consult traditional healers and use of traditional medicine.
**Problems experienced in seeking treatment**	
Travel long distance to access health facilities.	Patients travel overnight to reach the health facility
	Travel full day to reach a health facility.
	Critically ill patients die before reaching hospital.
The lack of money to pay for services and transport.	Not enough money to cover transport and treatment fees.

HEW, health extension worker; NGO, nongovernmental organisation.

The age of respondents plays an important role in the findings of this study. The study findings indicate that more than two-thirds (68.5%) of respondents were among the age group of childbearing women, and all acquired the mosquito nets through national free net campaigns. Similar findings were also identified in other countries.^20,21^ The majority of respondents were women (72.3%), and the participants for interviews were also dominated by women. Similar findings were shown in a study conducted in Cameroon.^20^ Culturally, care roles are linked to women, so malaria prevention was seen as a role for women; therefore, gender played a role in determining men’s participation in this study. The findings of this study revealed that men perceived women, children and the elderly as more vulnerable and at risk to malaria. This finding concurs with a study in southern Tanzania.^10^

Participants perceived that traditional and cultural methods of malaria prevention were widely available and accepted the use of herbs and plants, as indicated by the participants, which was supported in the literature as self-treatment^22^ is an alternative to unaffordable biomedical care services.^22,23,24,25^ The use of traditional medicine is popular not only in Namibia but also in other parts of the world.^2,26^ In the current study, participants used elephant and domestic animal dung, roots, leaves, branches, barks, wood of tumbleweeds, bitter bush and blue bush, marula fruit skin and fire.

The low level of knowledge among HEWs regarding traditional practices was one of the influencing factors that instilled fear among the participants about revealing traditional practices to members from health care institutions, as indicated by 8% of participants, which suggests that some participants did not reveal these practices because of fear. Individuals may hide and keep traditional practices secret because these are often stigmatised and labelled as inferior practices.^2^ In some cases, traditional practices are criticised as harmful and regarded as a threat to human health.^25^

While traditional practices are perceived as low-quality interventions, rural communities are struggling with money to buy and to use the best malaria prevention methods, such as long-lasting treated nets. Some participants revealed that they were encouraged to use only scientifically proven Western prevention practices such as mosquito nets, despite the minority of respondents (13.6%) being able to afford mosquito nets. Biomedical Western health services are often unaffordable.^3,25^ The encouragement to use only Western prevention methods is subjective and associated with insufficient knowledge about sociocultural factors that influence malaria prevention.^27^ Health extension workers have sufficient understanding of the cultural aspects of rural communities; however, there were times when they displayed behaviour of which is aggravated by cultural shock,^7,28^ as well as limited cultural awareness and sensitivity.^29,30^ The side-by-side presentation of the quantitative and qualitative results is presented in Table 2.

This study has highlighted the importance of considering sociocultural issues that need to be dealt with in order to improve malaria interventions by integrating them with the existing health system. It is also important to share indigenous practices with donor organisations, so that when the latter formulate policies on malaria prevention, they consider the sociocultural and socio-economic contexts of the targeted rural communities.

## Conclusion

The findings revealed that there is a need for diverse prevention strategies and combining the use of traditional practices with Western prevention should be encouraged. This is especially so because the rural communities demonstrated that there are problems with the use of long-lasting treated bed nets and hanging them in traditional sleep huts. Modification in giving health advice is also needed so that this advice will be aligned with sociocultural practices to promote acceptance and correct use of long-lasting treated mosquito nets among the rural communities in the Ohangwena region.

The procedures of malaria care need capacity-building for health care providers so that trust in indigenous knowledge can be used in the absence of Western prevention methods and also in addition to other Western malaria prevention practices. The limited access to and cost of Western prevention methods minimise protection because of priority and resource allocations, but it could be mitigated with the use of locally available traditional prevention practices used for many years in curbing malaria.

The findings of this study do not determine at which level of training and policy formulation the integration of sociocultural prevention practices should be incorporated into the existing health care system. It was beyond the scope of this study to involve unemployed HEWs, because there were no sites for them to be interviewed.

This study has contributed to new knowledge by revealing the need to combine standard prevention with traditional prevention practices, as in the fight against malaria, focus is also needed on sociocultural malaria care and prevention. There is a need to conduct a similar study in all nine regions of Namibia, which are considered malaria endemic and at risk for its transmission. There is also a need to create an enabling environment and political will that promotes improvement in malaria care. Intensified research focusing on interventions that address sociocultural factors for the prevention of malaria is required. In addition, there is a need for further study to test the efficacy of traditional practices used and to promote the valuable knowledge in the indigenous field to be validated.
